# Activation of peripheral nerve fibers by electrical stimulation in the sole of the foot

**DOI:** 10.1186/1471-2202-14-116

**Published:** 2013-10-08

**Authors:** Ken Steffen Frahm, Carsten Dahl Mørch, Warren M Grill, Nathan B Lubock, Kristian Hennings, Ole Kæseler Andersen

**Affiliations:** 1 Department of Health Science and Technology, Center for Sensory-Motor Interaction, Aalborg University, Aalborg, Denmark; 2 Department of Biomedical Engineering, Duke University, Durham, NC, USA; 3 Departments of Neurobiology and Surgery, Duke University, Durham, NC, USA

## Abstract

**Background:**

Human nociceptive withdrawal reflexes (NWR) can be evoked by electrical stimulation applied to the sole of the foot. However, elicitation of NWRs is highly site dependent, and NWRs are especially difficult to elicit at the heel. The aim of the present study was to investigate potential peripheral mechanisms for any site dependent differences in reflex thresholds.

**Results:**

The first part of the study investigated the neural innervation in different sites of the sole of the foot using two different staining techniques. 1) Staining for the Na_v_1.7 antigen (small nociceptive fibers) and 2) the Sihler whole nerve technique (myelinated part of the nerve). No differences in innervation densities were found across the sole of the foot using the two staining techniques: Na_v_1.7 immunochemistry (small nociceptive fibers (1-way ANOVA, NS)) and the Sihler’s method (myelinated nerve fibers (1-way ANOVA, NS)). However, the results indicate that there are no nociceptive intraepidermal nerve fibers (IENFs) innervating the heel.

Secondly, mathematical modeling was used to investigate to what degree differences in skin thicknesses affect the activation thresholds of Aδ and Aβ fibers in the sole of the foot. The modeling comprised finite element analysis of the volume conduction combined with a passive model of the activation of branching cutaneous nerve fibers. The model included three different sites in the sole of the foot (forefoot, arch and heel) and three different electrode sizes (diameters: 9.1, 12.9, and 18.3 mm). For each of the 9 combinations of site and electrode size, a total of 3000 Aβ fibers and 300 Aδ fibers was modeled. The computer simulation of the effects of skin thicknesses and innervation densities on thresholds of modeled Aδ and Aβ fibers did not reveal differences in pain and perception thresholds across the foot sole as have been observed experimentally. Instead a lack of IENFs at the heel decreased the electrical activation thresholds compared to models including IENFs.

**Conclusions:**

The nerve staining and modeling results do not explain differences in NWR thresholds across the sole of the foot which may suggest that central mechanisms contribute to variation in NWR excitability across the sole of the foot.

## Background

The nociceptive withdrawal reflex (NWR) protects the body by withdrawing the limb from a potential damaging stimulus. This reflex has been the subject of substantial research both in humans [1-4] and animals [5,6] as the NWR excitability reflects spinal nociceptive processes. The NWR can be evoked using cutaneous electrical stimulation and assessed by surface electromyography from selected muscles in the lower limb [7-9]. Since the NWR has a modular organization [7,10], stimulation of a certain skin area will activate reflex activity in a group of synergistic muscles leading to withdrawal of the affected skin area. Consequently, the reflex receptive field (RRF) of a muscle is defined as the skin area from where nociceptive stimulation elicits a NWR in that muscle [6,7]. In humans, the RRF is typically assessed via electrical stimulation of several sites at the sole of the foot [1,7,9]. Research has shown that the RRF expands during chronic pain conditions caused by central sensitization [11], and thus the NWR can be used to probe central sensitization.

The purpose of the present study was to investigate potential mechanisms underlying differences in NWR threshold and sensation across different sites in the sole of the foot. The NWR is more difficult to evoke from some areas of the foot sole than others, and the heel region is especially difficult requiring higher stimulation intensities [7]. The perceived stimulus quality also varies across the sole of the foot [12]. These differences in NWR threshold and sensation could be related to differences in skin thickness or innervation density across the sole of the foot. For example, the epidermal layers are thicker in the heel region and could act as insulators to the electrical stimulus [12]. Although no differences in mechanoreceptor densities across the sole of the foot have been observed [13], the NWR is mediated by activation of thin myelinated Aδ nociceptors [1,4], and little is known about the innervation density for nociceptors across the sole of the foot. Therefore, it is of interest to determine the nociceptive innervation densities in the sole and whether they could contribute to site-specific variation in NWR thresholds [1]. In the present study Aδ fiber innervation density across the sole of the foot was visualized using an antibody to the Na_v_1.7 sodium channel, which is primarily expressed in small myelinated sensory afferents like the nociceptive Aδ fiber [14]. Further, the innervation by myelinated fibers was visualized using Sihler’s method.

Differences in nociceptor density and skin layer thickness must be considered together to further understand the topography of NWR activation. Previously, the combination of volume conductor and nerve models has successfully been used to investigate how the cutaneous stimulation activates the nerves [15,16]. These models focused on how different stimulation parameters (e.g., electrode size) affected the type of nerve fibers activated. The present study combined visualization of nerve fiber densities and mathematical models of nerve fiber activation to understand the mechanisms contributing to topographic differences in the NWR thresholds and the sensation across the sole of the foot.

## Methods

### Cutaneous nerve staining

Following approval from the Institutional Review Board at Duke University, two post-mortem foot specimens were obtained from The International Institute for the Advancement of Medicine (Jessup, PA, USA). The exclusion criteria were any form of neural disorder, diabetes, HIV/AIDS or BMI > 30. The feet were fixed in 10% formalin for one month. Following fixation two different staining techniques were used to visualize innervation across the sole of the foot.

#### Sihler’s method nerve staining

**Figure 1:**
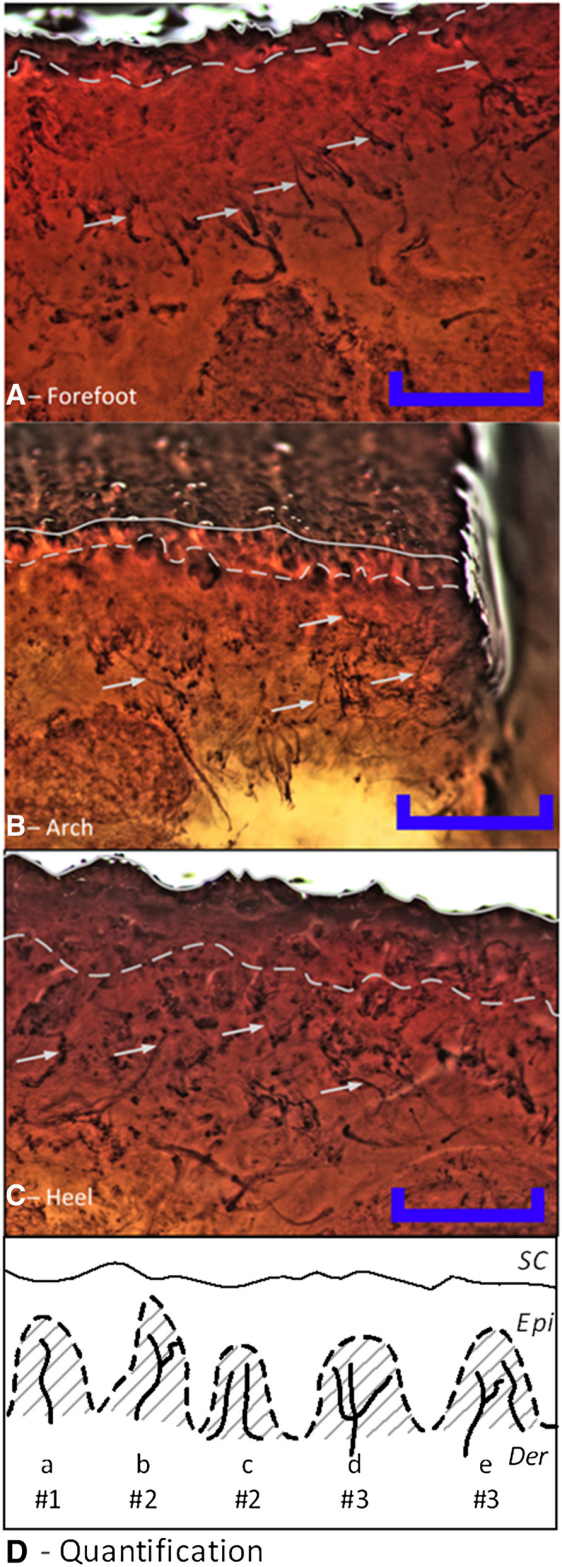
**Staining of cutaneous nerves across three sites on the sole of the foot using Sihler’s method.** Nerve fibers were stained in abundance across all three sites; **A** – Forefoot, **B** – Arch, **C** – Heel. The epidermis in the heel was thicker than in the forefoot and arch. Thus, the identifiable nerves terminated deeper in the heel. The solid line in **A-C** is the skin surface, the dashed line is the dermo-epidermal junction, and the arrows indicate examples of cutaneous nerve fibers used for quantification. **D***–* Approach used to quantify the number of nerve fibers. The # value indicates how many fibers were counted in each of the five examples. Na_v_1.7 immunoreactive nerve fibers were only counted inside the dermal papillary, the grey area in **D**, while nerves identified using Sihler’s staining were counted inside the dermis over a maximum distance of 1 mm from the dermo-epidermal junction. Scale bars represents 1 millimeter. SC: stratum corneum, Epi: Vital epidermis, Der: Dermis.

Sihler’s technique [17] was used to stain the myelin of the nerves in the sole of the foot. Innervation density at the heel, arch, and forefoot (under the distal part of the second metatarsal) was assessed (Figure 1). The thick skin at the sole of the foot acts an efficient barrier to chemicals, preventing the Sihler’s solutions from penetrating the tissue. To allow the solutions to penetrate the tissue, a grid was sectioned into the sole of the foot. The grid size on the skin surface was 10 mm × 10 mm. The grid was cut from the skin surface into the deeper hypodermis or muscle tissue (minimum depth 10 mm). Following the sectioning of this grid, the maceration was started. The maceration (immersion in KOH) took 5–6 weeks. Following the maceration the specimens were rinsed and decalcified in Sihler’s solution 1 (acetic acid solution [17]). The decalcification took 2–3 weeks. Following decalcification the specimens were cut into blocks based on the previously sectioned grid. The blocks were cut from the rest of the foot as deep as possible; the minimum depth was 10 mm. These tissue blocks were then rinsed and stained in Sihler’s solution 2 (Ehrlich's hematoxylin solution [17]) for 1–2 months. During the staining the tissue was gently agitated (80 rpm). The tissue was destained (Sihler’s solution 1 with agitation) and neutralized (lithium carbonate solution with agitation). Finally, the stained and neutralized specimens were examined under a dissection microscope.

#### Sodium channel Na_v_1.7 staining

After fixation, two punch biopsies were taken from each foot at the forefoot, arch and heel from the sole and from the dorsum of the foot using a 4 mm biopsy needle inserted perpendicularly to the skin surface. The fixed biopsies were cryoprotected in 30% sucrose, placed in a -80°C freezer for at least 48 h and subsequently longitudinally sectioned (from skin surface towards the hypodermis) at 50 μm on a cryostat.

The samples were placed in H_2_O_2_ for 10–15 min at room temperature to quench endogenous peroxidase activity [18]. After five rinses in phosphate-buffered saline (PBS), the samples were placed in a blocking solution of 0.01% avidin, 8% normal goat serum, and 0.1% Triton-X in PBS for 1 h at 4°C [19]. After three PBS rinses, the samples were placed in 1 mM EDTA pH 8.0 and placed in a temperature controlled oven at 80°C for 20 min to retrieve antigens after fixation. If the samples exhibited signs of shrinkage, they were removed from the oven. Following two PBS rinses, the samples were incubated overnight at 4°C in PBS with 0.005% biotin, 2% normal goat serum, and the primary antibody (1:1000, rabbit anti-Na_v_1.7, Alomone Labs, Jerusalem). Following four rinses in PBS the samples were incubated with the secondary antibody (1:200, anti-rabbit IgG, Vectorlabs, Burlingame, CA), and 2% normal serum buffered in PBS at 4°C for 1 h. Following four rinses in PBS the samples were incubated with ABC (Vectastain, Vector Labs, Burlingame, CA) for 1 hour at 4°C. After four rinses in PBS the samples were placed in DAB (Diaminobenzidine) solution for 3 minutes, after which the samples were rinsed with PBS and mounted on Superfrost+ slides and dried overnight. The slides were coverslipped using Permount mounting solution. Finally, the mounted sections were examined under a light microscope.

#### Quantification of the stained nerve fibers

The nerve fiber quantification technique was developed based on the methods used by [20] for counting intraepidermal nerve fibers (IENFs) and counts were based on the number of identifiable branches (Figure 1D). A single nerve fiber was counted as 1, a single fiber with two branches (e.g., a y-shape) was counted as 2, and for each additional branch the number was increased by 1 (e.g., a w-shape was counted as 3). The dermo-epidermal junction was easily identifiable in the specimens stained using Sihler’s method, and the nerves were quantified in the area between this junction and 1 mm into the dermis (Figure 1A-C). For the Sihler’s staining, a minimum of 3 sections were examined and quantified for each site. The Na_v_1.7 reactive nerves were only quantified inside the dermal papillae. The intrapapillary area was defined as the part of the dermis which bulged into the epidermis, illustrated as the grey areas in Figure 1D. For the Na_v_1.7 staining, a minimum of 15 sections were examined and quantified for each site.

### Finite element modeling

**Figure 2:**
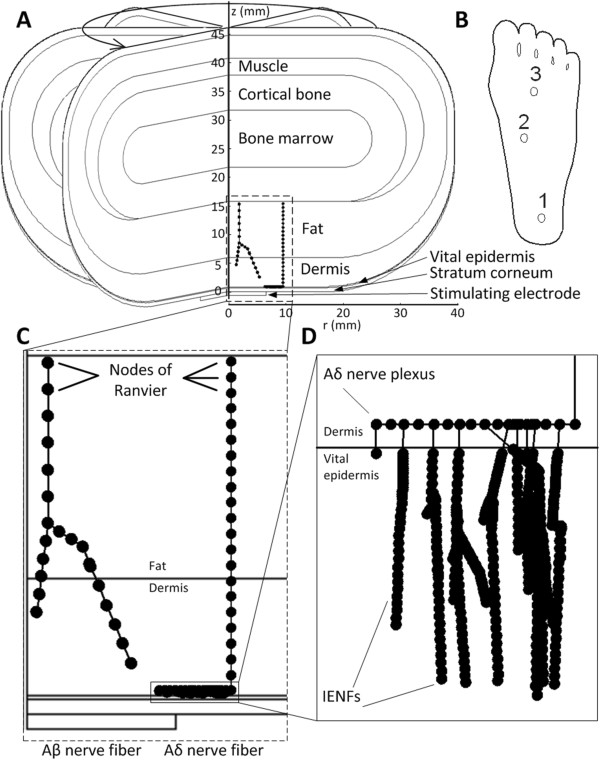
**Geometry of the finite element model (FEM), and examples of the morphology in the nerve model. A***–* The model was rotationally symmetric about the z-axis. The dorsum of the foot is the top of the model and the sole is the bottom. The geometry shown is for site 1. **B **– The location of the three modeled sites on the sole of the foot. **C***–* Examples of the morphology of one Aβ fiber and one Aδ fiber. The fiber morphologies were randomly generated. The nodes of Ranvier are indicated by the filled circles, but the diameters of the fibers are not to scale. Note the larger internode length for the Aβ fiber. **D***–* Detailed view of the morphology of the Aδ nerve plexus and intraepidermal nerve fibers (IENFs). The internode length for the IENFs was reduced to 1 μm to simulate the loss of myelin in the epidermis. Note that the aspect ratio of **D** was not maintained to improve visualization of the IENFs.

A two-dimensional rotationally symmetric model was implemented using the finite element method (FEM; COMSOL Multiphysics, Sweden) to calculate the extracellular potentials created in the tissue during electrical stimulation of the sole of the foot (Figure 2). The model comprised seven different subdomains representing the bone marrow, cortical bone, muscle, fat layer, dermis, vital epidermis, and stratum corneum (SC). The inner boundary of all the domains was placed on the vertical symmetry axis, located at *r* = 0, while the outer boundary of each of the subdomains was rounded to provide a simplified representation of the foot (Figure 2). The dimensions and electrical properties of the subdomains are listed in Table 1. The width of the innermost bone marrow was 25 mm. The normal experimental NWR setup includes abrading the skin and the use of gel electrodes, both of which will increase the conductivity of the SC. Thus, the conductivity of SC was increased in the model by a factor of ten, as compared to the literature [21]. In this way the impedance in the model fit previous experimental data [12]. The vital epidermis and dermis were modeled as being anisotropic [22] while all other layers were modeled as isotropic. The stimulation electrode was modeled at the lower boundary to represent an electrode on the sole of the foot. Three different sizes of stimulation electrodes were modeled with diameters of 9.1, 12.9, and 18.3 mm. The reference was placed on the upper boundary to represent a large reference electrode positioned on the dorsum of the foot [7]. Three different sites on the sole of the foot were modeled (Figure 2): site 1 was the heel, site 2 was the medial arch, and site 3 was the medio-central forefoot.

**Table 1:** Electrical and geometric parameters in the model

	**Electrical properties**		**Source**	**Thickness (mm)**				**Source**
				**Location (sole/rest of foot)**				
Tissue					Heel	Arch	Forefoot	
**Bone**	ϵ_r_	10,000	[23]	Sole & rest	10	10	10	
**marrow**	σ (S/m)	0.09						
**Cortical**	ϵ_r_	3,000	[23]	Sole & rest	6	6	6	[15]
**bone**	σ (S/m)	0.02						
**Muscle**	ϵ_r_	1.5×10^6^	[24]	Rest	3	3	3	[25]
	σ (S/m)	0.5		Sole	0	2	0	US ^*^
**Fat**	ϵ_r_	10,000	[24]	Rest	4	4	4	[16]
	σ (S/m)	0.025		Sole	9.7	5.3	6.1	US
**Dermis**	ϵ_r_ – *r*	1.26×10^6^	[22]	Rest	1.3	1.3	1.3	[16]
	ϵ_r_ – *z*	4.01×10^5^						
	σ (S/m) - *r*	2.57		Sole	5.1	4.3	4.6	US-SB ^**^
	σ (S/m) - *z*	1.62						
**Vital**	ϵ_r_ - *r*	2.71×10^5^	[22]	Rest	0.042	0.042	0.042	SB
**epidermis**	ϵ_r_ - *z*	7.30×10^4^						
	σ (S/m) - *r*	0.95		Sole	0.213	0.094	0.107	SB
	σ (S/m) - *z*	0.15						
**Stratum**	ϵ_r_	1,500	[21]	Rest	0.033	0.033	0.033	SB
**corneum**	σ (S/m)	5×10^-4^		Sole	0.748	0.161	0.253	SB

The relative permittivity (ϵ_r_) and conductivity (σ) were adopted from literature. The geometry values for the sole of the foot were determined from ultrasound (US) scans and the skin biopsies (SB) used for the Na_v_1.7 immunochemistry. Since the US scans provided only a bulk measure for the entire skin, and the SB provided only an estimate of the thickness of the stratum corneum and vital epidermis (*), the thickness of the dermis was calculated as the thickness of the entire skin subtracted the thickness of the entire epidermis (**, Stratum corneum + Vital epidermis).

### Nerve modeling

A cable model of a nerve fiber was combined with the FEM to estimate the stimulation threshold of Aβ and Aδ fibers innervating the skin of the foot sole. Even though the NWR is not mediated by Aβ fibers these were modeled to enable a comparison between perception and pain thresholds. The nerve fiber model was based on a simplified version of the McNeal model, as described in [16]. The morphology of the branches was created using a random generator as done in [16]. All fibers were initiated at the border of the fat layer farthest away from the skin surface at a randomized distance of 0 to 14 mm from the symmetry axis (Figure 2). The initial diameters for the Aβ and Aδ fibers were 9 and 4 μm, respectively. The ratio between fiber diameter and internodal length was 100, the resistivity of the axoplasm was 1.1 Ω*m, the nodal membrane capacity was 0.02 F/m^2^, and the nodal membrane conductance was 304 S/m^2^ for both Aβ and Aδ fibers as used in the original McNeal model [26]. At each node of Ranvier the Aβ fibers had 10% chance of creating a new branch. Whenever an Aβ fiber branched, its diameter shrank by 5% (Figure 2) and the two new branches took a random direction relative to the original direction of the nerve fiber, however, always going in the direction of the skin surface.

To model the dermal nerve plexuses of cutaneous Aδ fibers [27], the morphology of the Aδ branch model was modified from the original model found in [16]. The basic morphology of the Aδ fiber consisted of a vertical stem which terminated at a horizontal plexus. The fiber diameter in the plexus was 3 μm. From this plexus smaller fibers sprouted towards the skin surface. The plexus was initiated at a randomized depth in the dermis between 0 and 100 μm below the dermoepidermal junction. When the nerves sprouting from the plexus were inside the epidermis, the internodal length was reduced to 1 μm to represent the loss of myelin and the fiber diameter was reduced to 1 μm [27]. The number of branches in the plexus was randomized between 14 and 21 per mm [28]. The termination depth of the IENFs was randomized inside the epidermis. If the fibers were about to continue into the SC, the branching was stopped.

The activation node for both Aβ and Aδ fibers was defined as the node of Ranvier with the largest depolarization of the membrane potential [16]. Due to differences in model geometry caused by thicker skin layers in this study, the number of simulated nerve fibers was modified from those used in [16] to model the lower nerve innervation density in the sole as indicated both in the current study and by [29]. To simulate approximately 15% lower innervation in the sole than in other skin areas as found by previous research [29], the number of simulated fibers was set at 3000 Aβ fibers and 300 Aδ fibers for each of the 9 combinations of different electrode location and electrode diameter.

During the simulations the stimulation pulse duration was set to 1 ms. The activation threshold in the model was defined as a depolarization of 20 mV for the 1 ms pulse as described in the original McNeal model [26].

### Data analysis and statistics

Following quantification of the nerve fibers, the results were examined for significant differences across the tested sites. For both Sihler’s and Na_v_1.7 a 1-way analysis of variance (ANOVA) was applied. The statistical analysis was completed in SPSS. If the ANOVA showed a significant main effect, a Tukey post-hoc test was applied. p-values of less than 0.05 were considered significant.

## Results

### Cutaneous nerve staining

**Figure 3:**
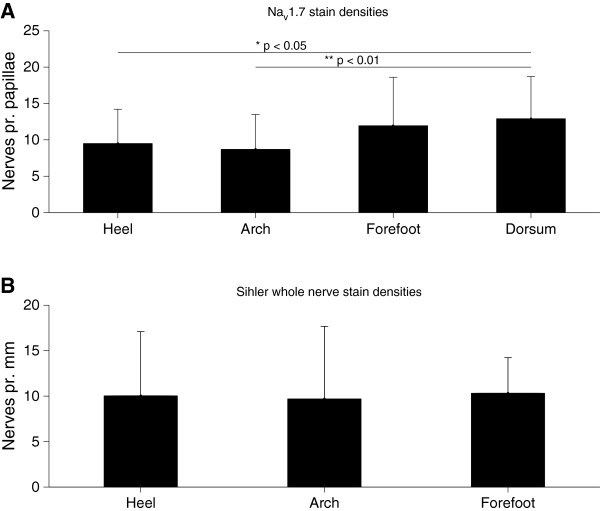
**Quantification of the identified nerves from Na**_**v **_**1.7 immunoreactivity and Sihler staining. A***–* Number of intrapapillary nerves per papillae showing Na_V_1.7 immunoreactivity. There were significant differences between the sites (1-way ANOVA, p < 0.01, F_(3,170_ = 5.174); the heel (post-hoc, p < 0.05) and arch (post-hoc, p < 0.01) had significantly lower nerve fiber densities than the dorsum. There were no statistically significant differences between the sites in the sole of the foot. **B***–* No significant differences in the densities of intradermal nerve fibers were found between the sites stained with Sihler’s method (1-way ANOVA, p=0.994, F_(2,8)_ = 0.006).

Application of Sihler’s method to post-mortem human feet revealed myelinated nerves in the dermis and hypodermis across the sole of the foot, but not in the epidermis. Larger trunks originated in the deep tissue and coursed toward the tissue surface as repeated branching resulted in smaller diameters (Figure 1). Quantification of the stained nerve fibers showed no significant differences between the sites across the sole of the foot (1-way ANOVA, p=0.994, F_(2,8)_ = 0.006*,* Figure 3).

**Figure 4:**
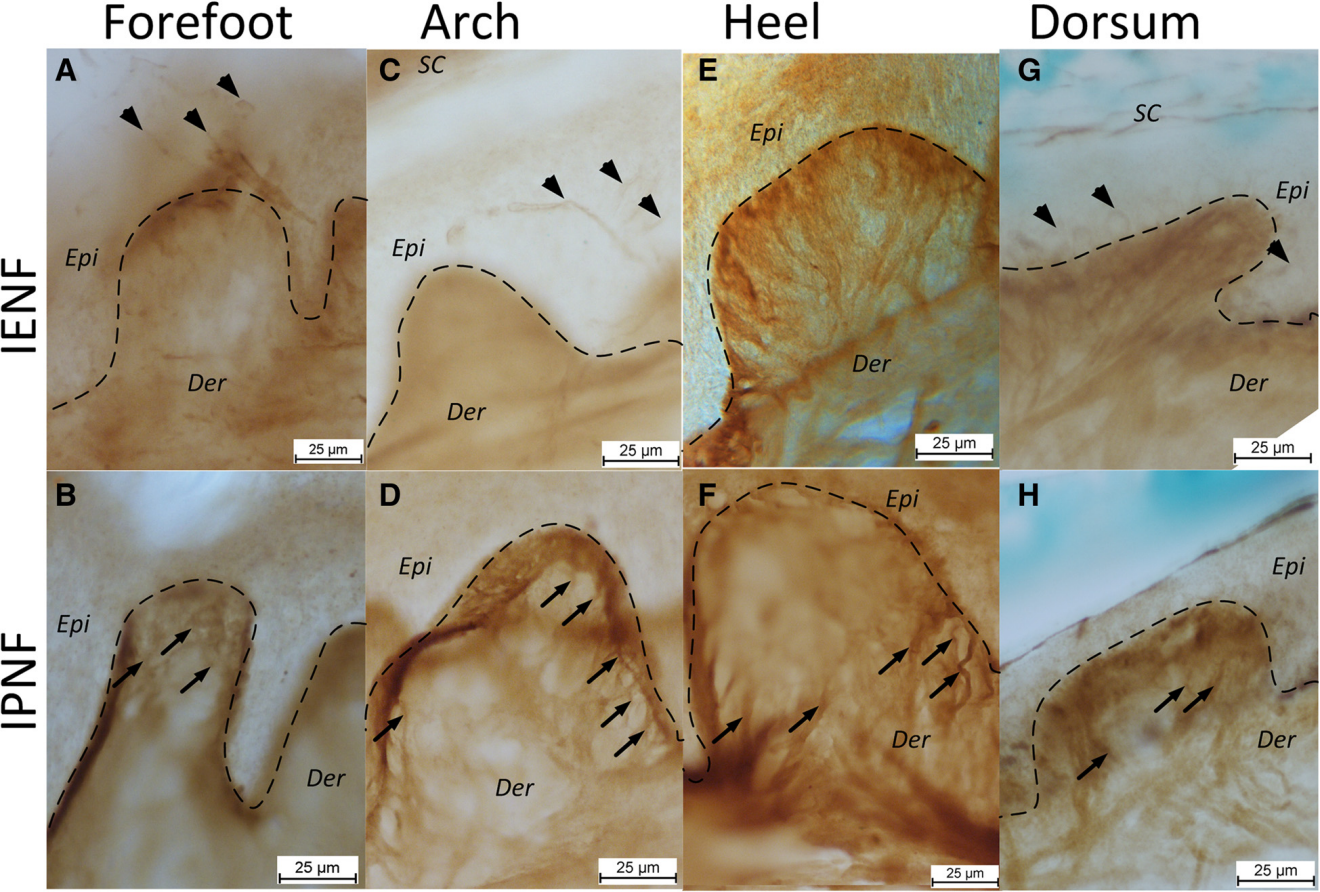
**Immunolabeling of cutaneous nerve fibers using Na**_**v **_**1.7 antibodies in different skin areas in the sole and dorsum of the foot.** The intra epidermal nerve fibers (IENF) were less abundant in the sole **(A,C,E)** than in the dorsum **(G)**. Intrapapillary nerve fibers (IPNF) were observed at all sites **(B,D,F,H)**. There were several sections where no IENFs were observed in the sole, while IENFs were present in the dorsum in most sections. IENFs were not observed in the heel. Arrowheads indicate examples of IENFs. Arrows indicate examples of IPNFs. SC: stratum corneum, Epi: Vital epidermis, Der: Dermis. The dashed lines indicate the dermo-epidermal junction.

Immunocytochemical staining of the Na_v_1.7 channel revealed identifiable nerve fibers in both the dermis and vital epidermis (Figure 4). Intrapapillary nerve fibers were very abundant in the dermal papillae and most fibers were oriented directly towards the top of the dermal papillae where some continued into the epidermis (Figure 4). However, no Na_v_1.7 immunoreactivity was identified inside the epidermis at the heel, and at this location there were only identified fibers in the dermal papillae. The density of Na_v_1.7 immunoreactive nerve fibers in the papillae was significantly lower at the heel (1- way ANOVA, F_(3,170_ = 5.174 with Tukey’s post-hoc, p < 0.05) and arch (1-way ANOVA, F_(3,170_ = 5.174 with Tukey’s post-hoc, p < 0.01) as compared to the dorsum (Figure 3). However, *within the sole* no significant differences in the density of Na_v_1.7 immunoreactive nerve fibers between sites appeared.

### FEM & nerve modeling

**Figure 5:**
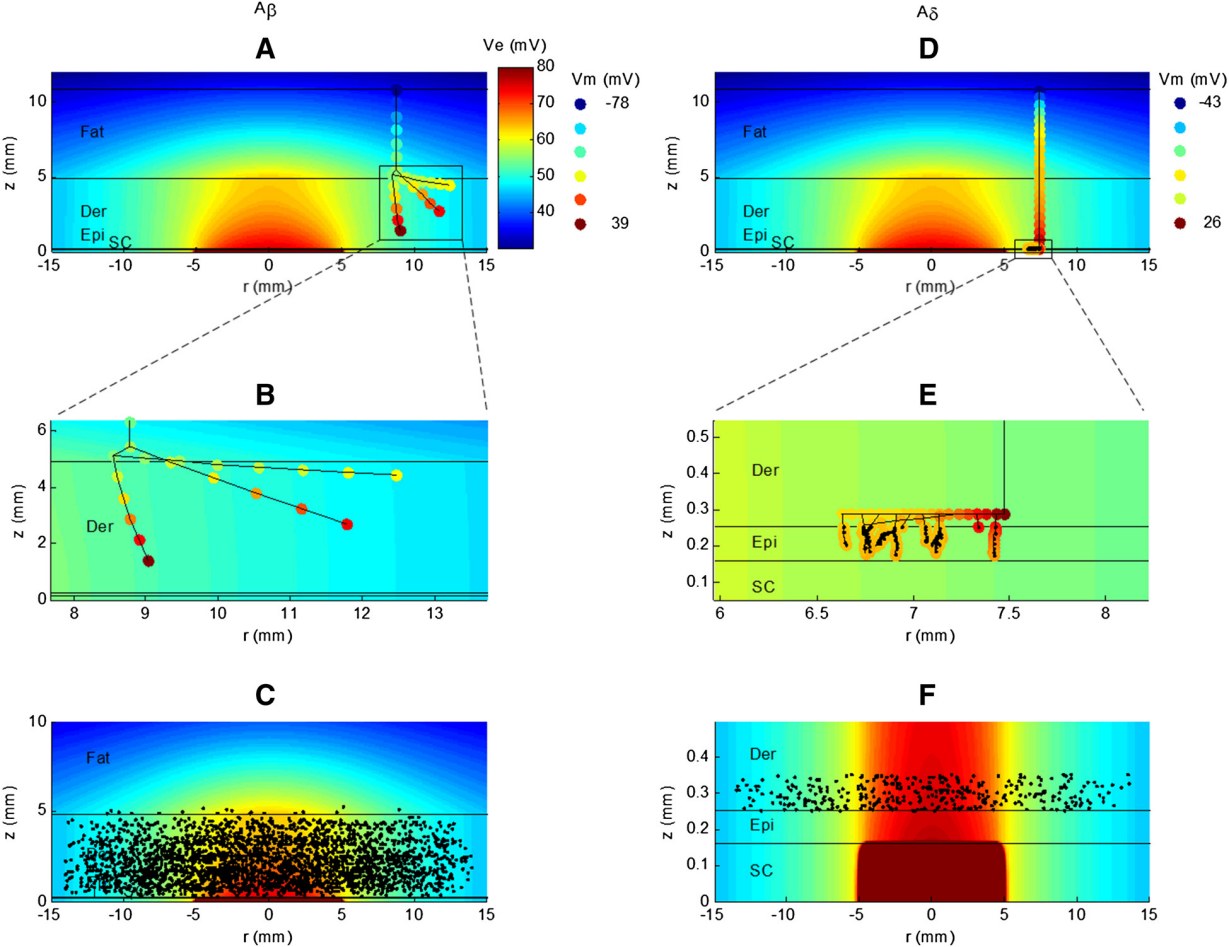
**Distribution of extracellular potential (Ve) in the finite element model and corresponding changes in membrane potential (Vm) in the nerve fiber model.** An example of both an Aβ fiber **(A-B)** and an Aδ fiber **(D-E)** are depicted. **B** and **D** are zoomed versions of **A** and **D**, respectively. The colored circles in **A,B,D,E** indicate the nodes of Ranvier in the models, and the colors indicate Vm. The stimulation current used to calculate Ve and Vm was 1 mA. Panels **C** and **F** illustrate the locations of the nodes of Ranvier (black dots) that were activated in the models for Aβ and Aδ fibers, respectively. In **C** it can be seen that most Aβ fibers are activated at nodes which lie inside the dermis. The activation of Aδ fibers **(F)** occurred in the plexus which is located at a random depth within the most superficial 100 μm of the dermis. SC = stratum corneum, Epi = vital epidermis, Der = dermis. The electrode diameter is 9.1 mm, and r = 0 indicates the vertical symmetry axis in the model. The horizontal black lines indicate the boundaries between the different tissue layers. The color scale for Ve was truncated to clarify the potentials in the vital epidermis and dermis. The model geometries were taken for site 2, the lateral arch (Table 1). Note that the aspect ratios in **B** and **E** are not maintained to improve visualization.

The mathematical model showed that Aβ fibers were most often activated in the node closest to the stimulating electrode (cathode) where the gradient of the extracellular electric field was largest (Figure 5A-C). In contrast, the Aδ fibers were activated at the node where the plexus met the vertical fiber stem (Figure 5D-F). Comparing the positions of the nodes where activation occurred, Aβ fibers were activated throughout the dermis (Figure 5A-C) while Aδ fibers were activated within the most superficial 100 μm of the dermis (Figure 5D-F).

**Figure 6:**
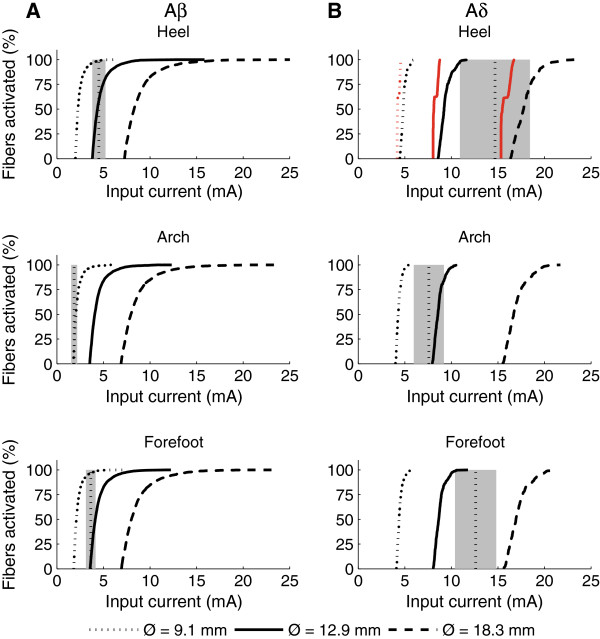
**Calculated stimulus–response curves for modeled Aβ and Aδ nerve fibers.** Generally Aδ fibers **(B)** had higher thresholds than Aβ fibers **(A)**. Three electrode sizes were modeled and the larger electrodes required larger stimulation currents to activate the nerve fibers. For the Aδ fibers at the heel, the red curves are the stimulus–response functions from models without intraepidermal nerve fibers (IENFs). The lack of IENFs decreased the activation threshold. The grey areas depict experimental values of perception and pain threshold (mean ± 95% confidence intervals). Perception thresholds are depicted with the Aβ fibers and the pain thresholds are depicted with Aδ fibers. Original data taken from [30], using an electrode diameter of 8 mm. Simulated pulse durations were set to 1 ms.

The stimulus–response properties of the neural model revealed clear differences between Aβ and Aδ fibers (Figure 6). As would be expected, the Aδ fibers had higher threshold than Aβ fibers seen as a right-wards shift of the stimulus–response curves (Figure 6).

The computed thresholds of Aβ and Aδ fibers were compared with previously published experimental values for perception and pain thresholds [30] obtained from 13 healthy subjects using a stimulating electrode diameter of 8 mm and several stimulation sites across the sole of the foot including the three sites tested in this study. The experimental values for the perception corresponded well with the thresholds of the smallest electrodes (Ø 9.1 mm) for the modeled Aβ fibers (Figure 6A). Similarly, the threshold of the Aδ fibers at the arch fitted well the experimental values for the pain threshold. However, at the heel and forefoot the computed Aδ fiber thresholds were 1.5-3 times higher than the experimentally determined pain perception thresholds (Figure 6B).

Removing the IENFs from the Aδ fiber model decreased the thresholds slightly (Figure 6B). Therefore, the apparent lack of IENFs at the heel does not seem to explain why NWRs are more difficult to evoke at this site compared to the rest of the sole of the foot. To investigate this further, the threshold for Aδ fibers with an intracellular current injected at the node where the nerve trunk branched into the plexus was determined with and without IENFs. With IENFs the activation threshold was 0.249±0.017 nA and without IENFs the activation threshold was 0.223±0.005 nA. Consequently, the removal of IENFs decreased the activation threshold by 9%.

## Discussion

This study documented different nociceptive nerve fiber innervation densities between the dorsum and the sole of the foot, but not across the sole of the foot itself, except an apparent lack of IENFs at the heel. However, neural modeling suggested that the lack of IENFs cannot explain the differences in perception and NWR thresholds between different sites on the sole of the foot. Furthermore, the modeling indicated that the differences in skin thickness across the sole of the foot were not a substantial contributor to the differences in neural activation either.

### Methodological considerations

Although several studies have used the PGP9.5 antibody to stain nerve fibers in the dermis and epidermis [18,20] including the sole of the foot [29], the PGP9.5 antibody does not discriminate between nerve fiber types. In humans, Aδ fibers but neither Aβ nor C fibers mediate the NWR. Therefore, the skin was stained for sodium ion channel Na_v_1.7 to more selectively visualize Aδ fibers. The nerve counts for the control staining from the dorsum of the foot are comparable to prior observations [29] indicating that the applied method is valid. The quality of the Na_v_1.7 immunoreactivity appeared to be higher in the dermal papillae than in the vital epidermis. Therefore, to eliminate the possibility of false negative findings, the dermal papillae were chosen for the investigation of innervation density.

Sihler’s whole nerve staining technique [17] revealed no differences in the deeper, myelinated innervation at the different sites. Sihler’s staining periods, as suggested by Mu and Sanders, had to be extended considerably since the skin and deeper tissues were thicker and denser on the sole of the foot than on the muscle tissues typically stained with this technique [17]. In particular, the maceration stage had to be extended from three to six weeks. Furthermore, the staining duration was also extended from four weeks to 1–2 months until nerves could be seen under a dissection microscope [17].

### Cutaneous nerve staining

Both staining techniques revealed identifiable nerves at all sites. The quantification of the nerve fiber densities revealed no significant differences within the sole of the foot. In contrast, the Na_v_1.7 stain did show differences between the dorsum of the foot and both the arch and heel area. However, the findings indicate that density differences in both the deeper and more superficial layers do not appear to be the basis of the different sensations and NWR excitability across the sole of the foot. The apparent lack of Na_v_1.7 responsive IENFs at the heel (Figure 4) is an interesting observation that could indicate a lack of Aδ fibers in the epidermal layers at the heel. This lack of IENF could be the consequence of the physical perturbations to the heel during gait. The lack of IENFs at the heel could cause noxious stimulation at the heel to activate fewer nociceptors as compared to other sites and was therefore further investigated using computer simulation of nerve fiber excitation.

The anatomical studies also showed that the thickness of the skin layers was very different across the sole of the foot (Figures 1 &3, and Table 1). The skin layers were thickest at the heel and thinnest in the arch of the foot. Since no IENFs are present in the SC [31], the thicker SC at the heel implies that any IENFs would be located further from the cathode. Similarly, a deeper dermo-epidermal junction will cause the intradermal and intrapapilary nerves to be located more deeply. Nerves located further from the cathode would be expected to require higher stimulus intensities to be activated.

### Neural modeling

For similar electrode sizes, the stimulus–response properties of the models of Aβ and Aδ fibers in general showed good agreement with experimental values for perception and pain thresholds, respectively (Figure 6). However, for the heel and forefoot there was a larger difference between the pain thresholds and the thresholds of modeled Aδ fibers. Furthermore, the activation of Aδ fibers occurred exclusively in the plexus where the Aδ fibers branched into their terminals (Figure 5). This finding is to be expected as the site of activation depends on the second order spatial derivative of the extracellular potential (activating function) rather than the absolute magnitude of the voltage [32]. The horizontal plexus of the Aδ fibers are myelinated and each node will be subjected to a significantly higher activating function than the unmyelinated nerve endings that branched from the plexus and terminated in the epidermis. Consequently, the morphology and myelination of the Aδ fibers in the plexus can explain why their activation occurred in the plexus rather than at the nerve endings in the epidermis, despite these being closer to the cathode.

The modeling results also indicate that the lack of IENFs at the heel cannot explain the differences in NWR thresholds. Lower activation thresholds in fibers without IENFs were to be expected from the finding that activation of Aδ fibers occurred in the plexus. Activation of a nerve fiber is the net result of a depolarizing contribution caused by the extracellular field and a hyperpolarizing factor caused by current redistribution through the intraaxonal space [33]. When the IENFs were removed, the load on the nodes of the Aδ fibers in the plexus decreased, and consequently, resulted in a lower activation threshold. This was confirmed by determining the activation threshold to intracellularly injected stimulation current at the node where the Aδ fibers branch out into the plexus. The fact that removing the IENFs decreased the activation threshold to intracellular stimulation confirmed that the IENFs had a significant influence on the node where activation occurred in the Aδ fibers.

### Possible central component

The results from the nerve staining and the nerve model indicated that the differences in perception, pain and NWR thresholds across the sole of the foot cannot be explained entirely by peripheral mechanisms. The small variations in the innervation densities across the sole of the foot fit well with the observation that NWRs could be evoked evenly across the foot in spinal cord injured subjects [34]. Moreover, frequent impact on the skin on the foot sole and upright posture could change how the central nervous system reacts to input from the heel. The input from the heel may be used primarily as postural feedback to ensure balance and assist gait, while mildly painful input may be inhibited to avoid perturbations and ensure continuous forward propulsion [35]. This gating would most likely reflect a central mechanism modulating the NWR, e.g., via presynaptic control.

## Conclusions

The present study revealed little or no differences in nerve fiber innervation densities across the sole of the foot. These minor differences and differences in skin thicknesses were not sufficient to explain the variation in perception, pain and NWR thresholds across the sole of the foot.

The use of graded electrode sizes for eliciting the NWR may provide a more localized current distribution activating the target nerve terminals and hence more localized input to the CNS. The use of smaller electrodes will also evoke a sharper sensation indicating a higher proportion of Aδ fiber activation [16]. On the other hand, the use of smaller electrodes leads to higher electrode impedances and hence a larger demand on the compliance voltage of the stimulators. Finally, the findings in this study suggest that the NWR modulation across the sole of the foot may reflect central mechanisms.

## Abbreviations

CNS: Central nervous system; DAB: Diaminobenzidine; EDTA: Ethylenediaminetetraacetic acid; FEM: Finite element method; IENF: Intra epidermal nerve fiber; IPNF: Intra papillary nerve fiber; Nav1.7: Voltage gated sodium channel 1.7; NWR: Nociceptive withdrawal reflex; PBS: Phosphate buffered saline; PGP9.5: Protein gene protein 9.5; RRF: Reflex receptive field; SB: Skin biopsy; SC: Stratum corneum; US: Ultrasound; Ve: Extracellular potential; Vm: Trans-membrane potential.

## Competing interests

The authors declare no competing interests.

## Authors’ contributions

KSF, WMG and OKA conceived and designed the nerve staining experiments. KSF and NBL performed the experiments. WMG contributed with reagents, materials and tools for the experiments. KSF, CDM and KH developed the mathematical models. KSF, CDM, WMG, KH and OKA analyzed the data. KSF drafted the manuscript. All authors have read and approved the final version of the manuscript.
